# Treatment of Occult Hepatitis C Virus Infection: Does It Need Special Attention?

**DOI:** 10.5812/hepatmon.16665

**Published:** 2014-07-01

**Authors:** Mohammad Saeid Rezaee-Zavareh, Behzad Einollahi

**Affiliations:** 1 Students’ Research Committee, Baqiyatallah University of Medical Sciences, Tehran, IR Iran; 2 Middle East Liver Diseases Center (MELD), Tehran, IR Iran; 3 Nephrology and Urology Research Center, Baqiyatallah University of Medical Sciences, Tehran, IR Iran

**Keywords:** Diagnosis, Clinical Laboratory Technique, Treatment Outcome, Hepatitis C Virus, Hemodialysis Unit

Dear Editor

Hepatitis monthly, as an esteemed journal, has published a very important and useful article, a meeting report, about therapeutic aspects of hepatic C virus (HCV) infection (1) in its previous issue. As you know, there is also a new form of chronic HCV infection called occult hepatitis C infection (OCI) that we would like to highlight some important points about it. OCI is defined by the detection of HCV-RNA in liver cells without presence of HCV-RNA and anti-HCV antibody in serum. It is also said that HCV-RNA can be found in the peripheral blood mononuclear cells (PBMCs) and in ultracentrifuged serum so that these finding can be used as a diagnostic method for OCI. Nonetheless, the gold standard method for diagnosis of OCI is finding HCV-RNA in hepatocytes via liver biopsy (2). It has been reported that OCI is usually milder that HCV infection. Although this infection can lead to minimal changes in liver, there are some reports of liver cirrhosis and subsequent hepatocellular carcinoma due to OCI (3). OCI has been seen in family members of OCI patients as well as in healthy population (3). It has also been described as a reason of cryptogenic liver disease (2). Furthermore, OCI has been reported in the patients on hemodialysis (4). Moreover, we know that controlling HCV infection in dialysis unit is very important and can be possible (5); however, considering OCI diagnostic method, the control of transmission seems more difficult. Some studies have investigated the clearance of OCI after treatment. In a prospective cohort study, Castillo et al. concluded that OCI can be persisted even after treatment in some cases and reported fluctuating changes in viremia level over time (6). In addition, Pardo et al. have evaluated the response to the antiviral therapy (pegylated interferon plus ribavirin) in patients with OCI. They have treated ten patients for 24 weeks and followed them up for 24 weeks after treatment; they reported more frequent HCV-RNA in PBMCs and abnormal alanine aminotransferase at the end of follow-up period than at the end of the treatment. Moreover, they detected HCV-RNA in hepatocytes of all cases (5 patients) who had undergone liver biopsy at the end of follow-up period and reported a sustained virological response in only 30% of cases. Ultimately, they concluded that treatment of patients with OCI with pegylated interferon plus ribavirin might have some benefits in these patients although OCI eradication might not be achieved in all cases (7). Existence of HCV-RNA in immune cells and liver tissue as its reservoir can lead to relapse or cause overt infection even after treatment (2); thus, this is one of the most important issues concerning treatment of OCI that must be taken into account.
